# Housing and Health

**Published:** 1921-01-22

**Authors:** 


### THE PUBLIC WELL-BEING.

### Housing and Health.

### ATTACKS ON THE MINISTRY.

Many foolish things are said and done in the name of economy, and one of the most foolish is the dead set that is being made in certain quarters against the Ministry of Health. That important but unlucky Department of State has for many months past been the direct object of attack of a certain type of newspaper and professional critic who steadily refuse to believe—as a fixed principle —that any good can emanate therefrom. We hold no brief for the Ministry, and we have ourselves, when occasion offered, criticised it, both in its policy and in details of administration. But there are notoriously two kinds of criticism; one is the honest and helpful kind, the other is the dishonest and damaging kind, which has for its motive the bringing of discredit by any means, fair or foul, upon the department or persons involved in the criticisms. The purely political motive we leave out of account; but it is surely time that a few disinterested journals should make a protest against these persistent attempts to deprive the Health Ministry of all power of usefulness by virulent attacks upon it at every conceivable opportunity, and by putting every kind of obstacle in the way of whatever policy it evolves or whatever scheme of administration it seeks to set up. This is an imperfect world, and the Health Ministry is an imperfect instrument for making it a better world; but, after all, it is the one State device we possess for stimulating activity in improving the national health, for carrying reforms into effect, and for giving a common purpose to local authorities and every variety of public health organisation. Deliberately to cripple and hamper its work, to cavil for the sake of cavilling, to seek to ridicule it out of existence, is, in our opinion, to commit an act of treachery against the public well-being.

The economists and the confessed enemies of the Ministry conspired only recently to deal a heavy blow, both at the Department and the public, by killing a measure which, with all its faults, contained a large number of provisions of great importance and real value to the community. In his housing policy Dr. Addison and his subordinates have received very little assistance from either the critics or the general public; and now a section of the Press is turning on him once again to rend him for the alleged crime of maliciously restricting the building of houses, though the house famine is almost as great as ever. The real facts of the situation are, as the writer learns on good authority, that the Treasury, in pursuance of the policy of the Government to cut down ruthlessly every State spending department, has instructed the Ministry that no schemes other than those which are already approved are to be proceeded with until further orders, and that Dr. Addison's Department has put forward a strong protest against this unexpected limitation. Dr. Addison is helpless in the matter unless the Treasury sanctions expenditure, and unless he can win over the Cabinet to his point of view. But it seems to us almost inevitable that the Treasury will have to modify its action in the near future. For a number of complicated reasons, our housing policy on its financial side has not been an outstanding success, but the need of houses is real and urgent; and until the present conditions of serious overcrowding, together with the presence of large slum areas which are going from bad to worse, are very greatly mitigated, industrial unrest and all the factors of serious disease will remain.

The point of view of the Treasury is supposed to be that by a policy of restriction there is good hope of a fall in the price of building materials which will substantially reduce the price per house when the time comes to sign a new batch of contracts. According to the latest returns, contracts have been signed for nearly 200,000 houses, while only about 21,000 have been completed, and it is argued, therefore, that ample work is in hand without entering upon fresh commitments. Moreover, the Registrar-General has estimated that only 140,000 new houses are required to restore the pre-war housing standard. This is a very astonishing statement, and it can be replied to in very definite terms. In the first place, the Prime Minister himself, as recently as December 21, admitted in the House of Commons that at least 500,000 houses were required to meet the legitimate needs of the population; in the second place, the Registrar-General's figures—whether accurately based or not—are dangerously misleading, because they ignore the disgraceful conditions of pre-war housing, and the serious depreciation and destruction of property that have been going on since the first year of the war; and in the third place, they will tend to have the deplorable effect of suggesting to local authorities that there is no reason for keeping up the housing campaign pressure at the very moment when the movement, after long delay, has gained just the impetus that was required.

It is stated that the Ministry of Health has had in its possession for over a year full information, supplied by local authorities throughout the country, as to areas which are being, or only have to be, dealt with as unhealthy under Part I. or Part II. of the Housing Act of 1890. Dr. Addison would be doing a good service if he were to publish this return immediately, for there is good reason to believe that it would startle the country, and, as a writer in the daily Press puts it, "do much to dissipate any illusion based upon the Registrar-General's housing figure." The figure, indeed, can easily be made to look foolish by the estimate made by local authorities in the Metropolitan Police district for 1919 that more than 100,000 houses were needed, and by the Lancashire local authorities that 90,000 were wanted in that county; so that in these two areas alone we are presented with a figure 90,000 in excess of that given by the optimistic Registrar-General.

